# Documenting research with transgender and gender diverse people: protocol for an evidence map and thematic analysis

**DOI:** 10.1186/s13643-017-0427-5

**Published:** 2017-02-20

**Authors:** Zack Marshall, Vivian Welch, James Thomas, Fern Brunger, Michelle Swab, Ian Shemilt, Chris Kaposy

**Affiliations:** 1 Social Development Studies and School of Social Work, Renison University College, University of Waterloo, Waterloo, Ontario Canada; 2 Division of Community Health and Humanities, Faculty of Medicine, Memorial University, St. John’s, Newfoundland and Labrador Canada; 3 Bruyère Research Institute, Ottawa, Ontario Canada; 4 School of Epidemiology, Public Health and Preventive Medicine, University of Ottawa, Ottawa, Ontario Canada; 5 EPPI-Centre, University College London, London, UK; 6 Health Sciences Library, Memorial University, St. John’s, Newfoundland and Labrador Canada

**Keywords:** Evidence map, Transgender, Gender diverse, Text mining, Research ethics, Responsible research, Research prioritization

## Abstract

**Background:**

There is limited information about how transgender, gender diverse, and Two-Spirit (trans) people have been represented and studied by researchers. The objectives of this study are to (1) map and describe trans research in the social sciences, sciences, humanities, health, education, and business, (2) identify evidence gaps and opportunities for more responsible research with trans people, (3) assess the use of text mining for study identification, and (4) increase access to trans research for key stakeholders through the creation of a web-based evidence map.

**Methods:**

Study design was informed by community consultations and pilot searches. Eligibility criteria were established to include all original research of any design, including trans people or their health information, and published in English in peer-reviewed journals. A complex electronic search strategy based on relevant concepts in 15 databases was developed to obtain a broad range of results linked to transgender, gender diverse, and Two-Spirit individuals and communities. Searches conducted in early 2015 resulted in 25,242 references after removal of duplicates. Based on the number of references, resources, and an objective to capture upwards of 90% of the existing literature, this study is a good candidate for text mining using Latent Dirichlet Allocation to improve efficiency of the screening process. The following information will be collected for evidence mapping: study topic, study design, methods and data sources, recruitment strategies, sample size, sample demographics, researcher name and affiliation, country where research was conducted, funding source, and year of publication.

**Discussion:**

The proposed research incorporates an extensive search strategy, text mining, and evidence map; it therefore has the potential to build on knowledge in several fields. Review results will increase awareness of existing trans research, identify evidence gaps, and inform strategic research prioritization. Publishing the map online will improve access to research for key stakeholders including community members, policy makers, and healthcare providers. This study will also contribute to knowledge in the area of text mining for study identification by providing an example of how semi-automation performs for screening on title and abstract and on full text.

**Electronic supplementary material:**

The online version of this article (doi:10.1186/s13643-017-0427-5) contains supplementary material, which is available to authorized users.

## Background

### Rationale

The aim of this review is to map and describe how transgender, gender diverse, and Two-Spirit (trans) people have been studied and represented within and across research in the fields of social sciences, sciences, humanities, health, education, and business. There is limited information about the scope of research focusing on trans individuals and communities. Because many people are not aware of the amount of research that has been conducted, this leads to misunderstandings and miscommunication. These beliefs are highlighted in statements by researchers such as “Limited empirical data are available regarding the mental health and general well-being of the transgender population” [1], “There is a dearth of health research about transgender people” [2], and “Literature regarding the gender variant population is very limited” [3]. Such misunderstandings may be particularly troublesome if trans community members are unaware of research that can potentially inform questions they have about their lives. Despite the lack of specific information, both researchers and community members have highlighted the links between research and the oppression of trans people [4–6]. Systematic research documenting the types of studies that have been conducted over time will provide details about the evidence that does exist and will help to identify opportunities for more responsible research [7] with gender diverse individuals and communities.

There are multiple challenges that restrict our ability to conduct reviews in the area of trans research. The first relates to the terminology used to describe transgender, gender diverse, and Two-Spirit people and the ways this influences search strategies. Language used to describe gender diverse people varies across stakeholder communities including medical diagnoses, terms used within or by communities, and phrases used across cultures and linguistic groups. As this terminology evolves over time [8], it adds to the number of terms that should be included in strong search strategies. A second challenge relates to subject headings, both in terms of the ways these headings reflect trans experience and their inability to remain up to date with language related to gender diversity [9]. These complications necessitate searches beyond subject headings, a process that is made more complex because it is difficult to search terms such as “trans” or “gender identity” by themselves due to the lack of specificity of these terms to the target study records and the consequent number of irrelevant results this produces. It is also necessary for search strategies to include both database-specific headings and independent search terms and to include terms such as mastectomy or vaginoplasty that may be relevant to both cisgender and transgender experience. The term cisgender refers to people who identify with the gender they were labelled at birth, also referred to as non-transgender people. Once searches are complete, screening is complicated by difficulties with identifying whether there are trans participants involved in the studies, or whether the articles are trans-focused, due to information that may be incomplete in the title and abstract. For example, these challenges arise when reviewing references that include trans people as part of larger studies with lesbian, gay, bisexual, trans, and queer (LGBTQ) communities, and surgery-related case reports.

Despite these difficulties, some researchers have attempted to raise awareness of the types of trans research available. One of the earliest examples is an annotated bibliography developed by Denny in 1994 [10]. Published in book format, this bibliography includes early articles, books, and community reports. Since then, we have also seen a slow increase in systematic reviews. Primarily focused in the area of trans health [11], researchers have conducted reviews related to gender dysphoria [12], HIV [13], cancer care [14], mental health [15], learning disabilities [16], support experiences and attitudes of parents of gender variant children [17], gender identity disorder in twins [18], and aging [19]. More commonly, we see trans studies included as part of larger reviews focusing on LGBTQ communities, men who have sex with men (MSM), or other marginalized populations (e.g., [20, 21]).

The proposed research, by incorporating an extensive search strategy, text mining, and evidence map, has the potential to build on knowledge in several fields. At this time, there are no evidence maps of trans research. By documenting this broad field of study, this review will increase awareness of existing trans research, identify evidence gaps, and inform strategic research prioritization [22]. Publishing the map online will also improve access to research for key stakeholders including community members, policy makers, and healthcare providers.

### Aim and objectives

The aim of this review is to map and describe how trans people have been studied and represented within and across multiple fields of research. The objectives are to:

1. Document trans research in the fields of social sciences, sciences, humanities, health, education, and business including information about study topic, sample demographics, and study design
2. Identify evidence gaps and opportunities for more responsible research with trans people
3. Assess the use of text mining for study identification
4. Increase access to trans research for community members, policy makers, and healthcare providers by establishing a web-based evidence map, including a searchable reference database.

## Methods

This protocol was prepared in accordance with the Preferred Reporting Items for Systematic Review and Meta-Analysis Protocols (PRISMA-P) [23] (see Additional file 1). An evidence map will be developed using the framework developed by Hetrick and colleagues [24] which includes four steps. Evidence maps are an emerging method [25] to “collate, describe, and catalog” knowledge across a broad subject area [26]. This information can then be leveraged by stakeholders to inform policy and clinical decisions [26].

### Eligibility criteria

As part of the process of developing evidence maps, it is recommended that researchers clarify concepts and engage key stakeholders in considering the potential scope of the review [27]. Accordingly, individual consultations were held with members of trans and cisgender communities to discuss terminology, search scope, and potential uses of an evidence map. Based on the results of consultations and pilot searches, the eligibility criteria were established to include all original research studies of any design, reported in English language peer-reviewed journals, that identifiably included trans people or their information, such as medical or surgical case reports with single participants, trans-focused qualitative or quantitative research, and population survey data that adequately identify trans or gender diverse participation.

### Information sources

Initial identification of potential databases was based on the goal of obtaining the broadest range of studies about trans people from multiple fields including social sciences, sciences, humanities, health, education, and business. A secondary emphasis was to gather research from countries and cultures around the world. For example, in order to properly capture research about gender diverse Indigenous people, three databases focused on Indigenous and First Nations research were included.

Once a draft list had been identified, overlap analysis of potential databases was conducted by a health sciences librarian [28–30]. Specifically, PubMed was chosen to capture the content not included in MEDLINE through Scopus [28]. Fifteen databases were selected to ensure the identification of diverse study designs [31] including Academic Search Premier, Anthropology Plus, Bibliography of Native North Americans, CINAHL, First Nations Periodical Index, Indigenous Studies Portal, LILACS, ProQuest Social Sciences Premium (contains Sociological Abstracts, ERIC, Social Services Abstracts & Applied Social Sciences Index and Abstracts), PsycINFO, PubMed, SciELO, Scopus, Social Work Abstracts, Web of Science, and Women’s Studies International.

### Search strategy

Search terms focus on transgender, gender diverse, and Two-Spirit identities and experiences. The search strategy is provided in Additional file 2. Because there are multiple terms used for (and/or by) trans people, and this language continues to shift over time [8], the full list of search terms is extensive and consists of terms related to gender identity (e.g., “trans woman”), diagnoses (e.g., “gender identity disorder” and “gender dysphoria”), medical and surgical procedures (e.g., vaginoplasty), terms used in a range of countries and cultures (e.g., hijra, waria, travesti), and language used historically (e.g., “transvestite”).

### Study records

#### Data management

The draft search strategy was reviewed with a health sciences librarian. Pilot searches were conducted in January 2015 for each search string in all 15 databases to ensure that the search was specific but not overly sensitive. Full searches were then conducted from January 25 to February 22, 2015, (see Table 1). After each search was complete, all references were imported to EndNote and subsequently imported into EPPI-Reviewer V.4.6.0.1 where duplicates were removed. EPPI-Reviewer is a web-based software designed to support the screening, data extraction, and data analysis phases of scoping and systematic reviews. Searches resulted in a total of 63,003 references. After removing duplicates, the total number of references included in the review is 25,242.

**Table 1:** Results of database searches

Database	*N* records
Academic Search Premier	9,477
Anthropology Plus	339
Bibliography of Native North Americans	75
CINAHL	2,386
First Nations Periodical Index	41
Indigenous Studies Portal	84
LILACS	738
ProQuest Social Sciences Premium	10,212
ProQuest Subject Terms	2,718
PsycINFO	6,223
PubMed	7,464
SciELO	482
Scopus	11,640
Social Work Abstracts	144
Web of Science	7,641
Women’s Studies International	3,320
Total number of references retrieved	63,003
Duplicates removed	37,761
Total number of references	25,242

#### Selection process

Abstracts will initially be screened based on the information in the title and abstract (level 1). References will be excluded if articles are not written in English, if they are not original research, if they do not include humans, or if they include only cisgender heterosexual people or people diagnosed with disorders of sex development (DSD). If a reference cannot be excluded at level 1, the full text of the article will be uploaded so that it can be screened more thoroughly (level 2).

##### Text mining

The large number of citations retrieved by electronic searches in such a complex and broad topic area inevitably creates workload challenges for reviewers who need to check them all for eligibility. The use of new technologies—text mining and machine learning—have been advanced as potential ways in which screening workload might be reduced [32]. When used in the context of reference screening in systematic reviews, a process known as “active learning” can be employed, whereby the machine “learns” from a relatively small sample of reviewer decisions and presents to the reviewer a set of references to screen next; the machine then learns from these screened references too, and the process continues in an iterative fashion. While effective at identifying the majority of relevant studies much earlier in the screening process than would otherwise be the case, there is a danger of the machine models becoming “over-fitted” early in the process, and some relevant studies not being identified. In order to reduce this risk, the citations are grouped together into thematically similar topics using topic modeling using Latent Dirichlet Allocation [33]; these topics can then be utilized as “features” within the machine learning process and also examined manually by reviewers in order to ensure that each topic has been adequately explored for potentially relevant studies.

##### Screening on full text

For full-text screening, two team members will review each reference, and any differences will be reconciled through discussion. Level 2 screening will identify original research that includes trans participants or their information. In addition, at this level we will identify studies that include only trans participants, research with photographs of trans people, research that includes trans participants as part of larger LGBTQ studies, and studies with both cisgender and trans participants. The purpose of identifying these details at level 2 is to support data extraction. After eligibility is confirmed based on a review of the full text, then the extraction of information from each article will begin (level 3).

#### Data collection process

Once all of the English-language peer-reviewed original research that includes trans people or their information has been identified, we will begin data extraction using a standardized data extraction form. The form will be piloted by two reviewers and then data extraction will be conducted by one person, with a second reviewer verifying data extraction results.

### Data items

Data extraction will focus on creating an evidence map emphasizing the extent and distribution [34] of trans research studies. The following information will be collected for mapping: study topic; study design, methods, and data sources; recruitment strategies; sample size and demographics (gender identity, sexual identity, race/ethnicity, age, geographic location, education, and income); terminology used to describe trans people; researcher name and affiliation; geographic location of data collection; funding source; and year of publication. Because we do not extract health-related outcomes, this evidence map has not been registered with PROSPERO.

### Data synthesis

#### Evidence map

In their recent systematic review, Miake-Lye et al. [25] highlighted the user-friendly formats of evidence maps, which often include graphs, visual figures, or a database that is searchable. For example, McCandless and Perkins [35] created an interactive infographic looking at the evidence for nutritional supplements. In addition, researchers including Snilstveit and colleagues [22] are contributing to gap maps that visually illustrate both evidence and gaps in research. With this project, the goal is to focus on mapping the information stakeholders are most interested in obtaining such as subject area, study design, and sample demographics. After data extraction is complete, information will be exported from EPPI-Reviewer into a database hosted by RSpace Repository at Renison University College, University of Waterloo (http://rspace.uwaterloo.ca/xmlui/). The initial plan is to incorporate an open access searchable database including title, abstract, and journal details, as well as information extracted as part of this evidence mapping process. Once the database has been populated, we will develop additional visually accessible tools that are more accessible to policy makers and community stakeholders, including the ability to combine searches using visual symbols, and to display information using formats such as bubble plots and color-coded summary tables.

## Discussion

This research will map and describe how trans people have been represented and studied within and across multiple fields of research. In addition to identifying the types of research that have been conducted, it will also provide information about which topics have been under-researched, who has been over- or under-included as research participants, and areas where further scoping studies or systematic reviews would be appropriate. Providing this information online will help to improve stakeholder access to research about gender diverse people and will contribute to increased knowledge democracy for transgender, gender diverse, and Two-Spirit individuals and communities. This study will also increase knowledge in the area of text mining for study identification by providing an example of how semi-automation performs for screening on title and abstract and on full text.

## Additional files

Additional file 1: PRISMA-P Checklist. Completed PRISMA-P Checklist including relevant source information. (PDF 54 kb)
Additional file 2: Sample search strategies. Search strategies for PubMed and Academic Search Premier. (DOCX 27 kb)

## Abbreviations

DSD: Disorders of sex development
HIV: Human immunodeficiency virus
LGBTQ: Lesbian, gay, bisexual, trans, and queer
MSM: Men who have sex with men
PRISMA-P: Preferred Reporting Items for Systematic Review and Meta-Analysis Protocols
Trans: Transgender, gender diverse, and Two-Spirit
