# Supplementary material for: Documenting research with transgender and gender diverse people: protocol for an evidence map and thematic analysis
Source: Syst Rev. 2017 Feb 20;6:35. doi: 10.1186/s13643-017-0427-5 (PMC5319144; doi:10.1186/s13643-017-0427-5)
Supplement: Additional file 1: — PRISMA-P Checklist. Completed PRISMA-P Checklist including relevant source information. (PDF 54 kb) [file 13643_2017_427_MOESM1_ESM.pdf]

PRISMA-P (Preferred Reporting Items for Systematic review and Meta-Analysis Protocols) 2015 Checklist

Section and topic	Item No	Checked	Source
**ADMINISTRATIVE INFORMATION**			
Title:			
Identification	1a	✓	Title page
Update	1b	NA	
Registration	2	NA	
Authors:			
Contact	3a	✓	Title page
Contributions	3b	✓	Page 14
Amendments	4	NA	
Support:			
Sources	5a	✓	Page 14
Sponsor	5b	NA	
Role of sponsor or funder	5c	✓	Page 14
**INTRODUCTION**			
Rationale	6	✓	Page 4-6
Objectives	7	✓	Page 6-7
**METHODS**			
Eligibility criteria	8	✓	Page 7-8
Information sources	9	✓	Page 9
Search strategy	10	✓	Page 9
Study records:			
Data management	11a	✓	Page 9

Selection process	11b	✓	Page 9-11
Data collection process	11c	✓	Page 11
Data items	12	✓	Page 11-12
Outcomes and prioritization	13	NA	
Risk of bias in individual studies	14	NA	
Data synthesis	15a	NA	
	15b	NA	
	15c	NA	
	15d	✓	Page 12
Meta-bias(es)	16	NA	
Confidence in cumulative evidence	17	NA	

NA – Not applicable
